# Combining the Physical Adsorption Approach and the Covalent Attachment Method to Prepare a Bifunctional Bioreactor

**DOI:** 10.3390/ijms130911443

**Published:** 2012-09-12

**Authors:** Mengxing Dong, Zhuofu Wu, Ming Lu, Zhi Wang, Zhengqiang Li

**Affiliations:** Key Laboratory for Molecular Enzymology and Engineering of the Ministry of Education, College of Life Sciences, Jilin University, Changchun 130012, China; E-Mails: dong.meng.xing@163.com (M.D.); wuzf06@mails.jlu.edu.cn (Z.W.); luming@jlu.edu.cn (M.L.); wangzhi@jlu.edu.cn (Z.W.)

**Keywords:** adsorption, amino-functionalized mesoporous silica, covalent attachment, myoglobin, lysozyme, peroxidase activity, antibacterial activity

## Abstract

Aminopropyl-functionalized SBA-15 mesoporous silica was used as a support to adsorb myoglobin. Then, in order to avoid the leakage of adsorbed myoglobin, lysozyme was covalently tethered to the internal and external surface of the mesoporous silica with glutaraldehyde as the coupling agent. The property of amino-functionalized mesoporous silica was characterized by N_2_ adsorption-desorption and thermogravimetric (TG) analysis. The feature of the silica-based matrix before and after myoglobin adsorption was identified by fourier transform infrared (FTIR) and UV/VIS measurement. With o-dianisidine and H_2_O_2_ as the substrate, the peroxidase activity of adsorbed myoglobin was determined. With Micrococus lysodeilicus as the substrate, the antibacterial activity of covalently tethered lysozyme was measured. Results demonstrated that the final product not only presented peroxidase activity of the myoglobin but yielded antibacterial activity of the lysozyme.

## 1. Introduction

In recent years, the enzyme has been employed in numerous applications in the form of immobilized enzyme preparation [1–3]. By nature, the immobilization of the enzyme is divided into two groups. (I) When the enzyme is immobilized on the outer surface of a support and hence directly exposed to the solution, the enzyme is easily in contact with the substrate, which may result in high catalytic efficiency in a catalytic reaction. On the other hand, the enzyme molecular is sensitive to adverse effects of some reagents in the solution, which may cause the loss of the activity of the enzyme. (II) In contrast, for the enzyme which is embedded in the support and finitely contacts with the solution, the catalytic activity of the enzyme in the support is only observed if the diffusion of the product and the substrate in the support is similar to the case in the solution [4].

In order to prevent the enzyme from direct exposure to the solution, some methods such as entrapment in semi-permeable membrane [5], sol-gel method [6] and physical adsorption [7] have been adopted. The encapsulation of the enzyme which possesses relatively large spatial size is often achieved by the semi-permeable membrane and sol-gel method. In the semi-permeable membrane approach, low mechanical stability of the membrane results in poor reusability of immobilized enzyme. In the sol-gel method, the support presents poor mechanical stability and the pore size distribution cannot easily be controlled. These disadvantages block practical application of immobilized enzyme in industry. In 2006, the encapsulation of fumarase, trypsin, lipase and porcine liver esterase was fulfilled through the “fish-in-net” approach in our experiment [8]. Taking into account the problem that three-dimensional size of the enzyme might be bigger than the pore size of the silica matrix, the enzyme and performed precursors were placed in a non-denaturing environment simultaneously to complete the encapsulation of the enzyme. The encapsulated enzyme presented perfect enzymatic activity and excellent reusability. The silica-based matrix, acting as the support of the enzyme, simultaneously possessed an ordered mesoporous structure and regular macroporous structure. Such technology has been used in practical applications such as the hydrolysis of lactose in the preparation of low lactose milk [9] and the removal of aniline in industrial wastewater [10].

When the spatial size of the enzyme molecule is smaller than the pore size, many kinds of mesoporous molecular sieves are used as support to achieve immobilization through physical adsorption. Due to their high mechanical stability, good chemical stability, large special surface area and pore volume, a silica-based matrix such as MCM-41 [11], SBA-15 [12] is widely applied to the adsorption of enzyme. Adsorbed enzyme preparations are easily separated from the reaction system and possess perfect reusability. Attributed to the merits mentioned above, adsorbed enzyme in mesoporous silica materials is suitable for industrial production.

However, the change of temperature, pH value and ionic strength can alter the surface charge property of the absorbed enzyme and mesoporous silica, leading to the disappearance or the decrease of translational and rotational restriction of the enzyme molecule after absorption [13]. Thus, the leakage of the adsorbed enzyme molecules occurs.

**Figure 1:**
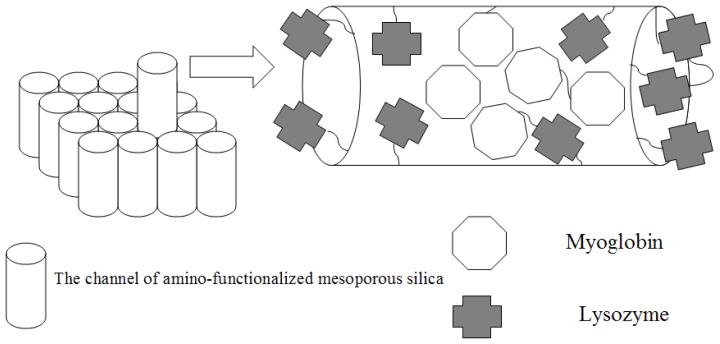
Schematic representation of the principle. First, the myoglobin was adsorbed into the channel of amino-functionalized mesoporous silica. Second, the lysozyme was linked to the external and internal surface of amino-functionalized mesoporous silica. The resulting product possessed two different enzymatic activities.

In order to overcome the leakage problem, two-step immobilization was carried out to achieve the immobilization of the enzyme in this experiment. Herein, myoglobin and lysozyme were selected as a model protein owing to its well known physicochemical property and commercial availability. First, amino-functionalized mesoporous molecular sieve was used to absorb myoglobin. Second, lysozyme was linked to the amino group of the amino-functionalized mesoporous molecular sieve to avoid the leaching of the absorbed myoglobin. Finally, the bifunctional bioreactor was obtained (Figure 1).

## 2. Results and Discussion

### 2.1. Characterization of Amino-Functionalized Mesoporous Silica

The aminopropyl-functional SBA-15 mesoporous material was first synthesized by one-pot co-condensation synthesis in 1996 [14]. The amino-functionalized mesoporous silica produced by co-condensation synthesis possessed higher loading of the functional group [15]. The co-condensation synthesis provided more homogenous distribution of organosilane functionalities without collapse of the mesoporous framework [16]. In 2005, the conventional co-condensation method was improved by a prehydrolysis-step of the silica resource before the adding of APTES [17]. In this experiment, the improved co-condensation synthesis was adopted to simplify the synthesis procedure and prepare the desired amino-functionalized mesopcorous silica.

**Figure 2:**
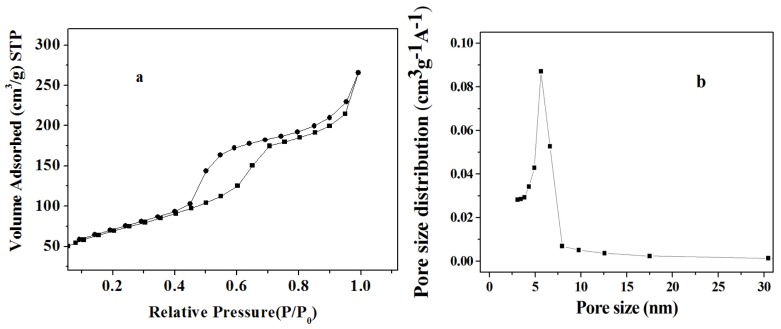
(**a**) Nitrogen adsorption-desorption isotherms and (**b**) BJH **(**Barrett-Joyner-Halenda) pore size distribution plots of amino-functionalized mesoporous silica synthesized using the co-condensation method.

As suggested by Soofin *et al.*, the mesoporous ordering of amine-silane decreased with the increasing molar composition of APTES in the synthesis system [17]. When the molar composition was close to 0.2, X-ray diffraction peaks of amine-silane began to weaken. In order to simultaneously acquire the maximum loading of amino group and ordered mesostructure, 0.2 was chosen as the molar composition of the APTES in this experiment. The sample showed the H_1_ type hysteresis loop (Figure 2a). The pore size distribution of the sample was very sharp (Figure 2b). The BET surface area, pore volume and BJH pore diameter were 250 m^2^/g, 0.41 cm^3^/g and 5.7 nm, respectively. It is necessary that the pore size should be larger than the molecular size of the enzyme to ensure successful adsorption [18]. Furthermore, when the size of the mesoporous material matches the dimension of the enzyme molecule, the enzyme is deeply absorbed into the internal surface of mesoporous material rather than on the external surface [7,19]. Myoglobin (*M*_r_ 16700) contains a single polypeptide of 153 amino acid residues with one molecule of heme as a prosthetic group. The myoglobin possesses a molecular dimension of 4.5 nm × 3.5 nm × 2.5 nm [20]. Obviously, amino-functionalized mesoporous silica synthesized in this experiment was suitable for the adsorption of myoglobin.

**Figure 3:**
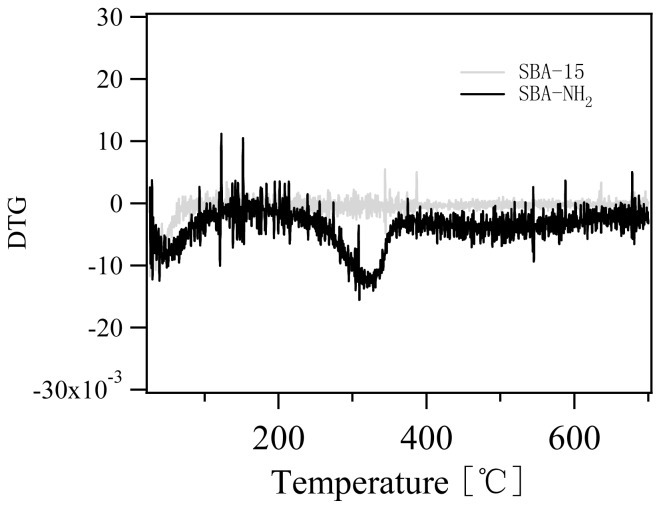
DTG (Differential thermogravimetry) profile of amino-functionalized mesoporous silica (black line) and SBA-15 (gray line).

The DTG profile of amino-functionalized mesoporous silica showed two peaks at 50 °C and 320 °C, respectively (Figure 3). The evaporation of physically adsorbed water gave rise to the weight loss at temperature lower than 100 °C. The decomposition of aminopropyl group of amino-functionalized mesoporous silica resulted in weight loss at 250 °C–500 °C [13]. Moreover, either the dehydroxylation of the Si-OH group or the elimination of the residual ethoxy group originated from incomplete hydrolysis of silicon ethoxide might lead to the slight weight loss above 500 °C [21–22].

**Figure 4:**
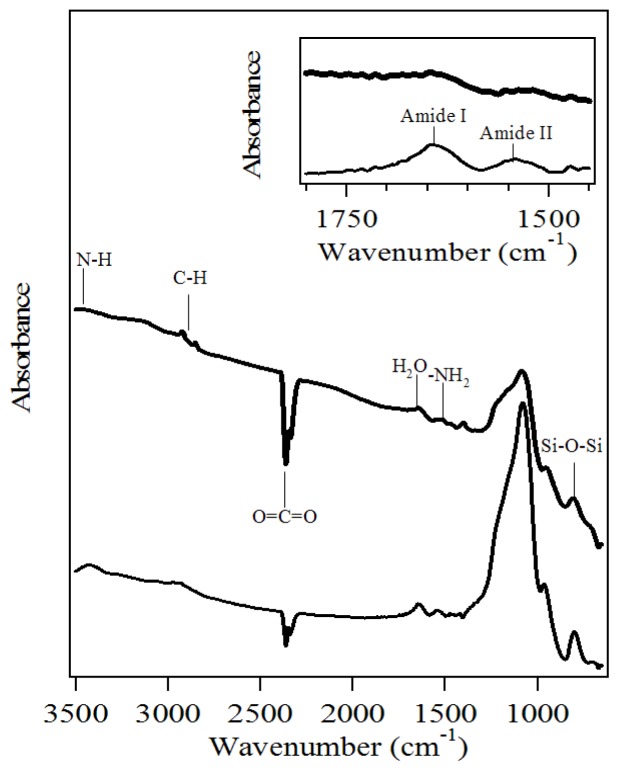
Fourier transform infrared (FTIR) spectra of amino-functionalized mesoporous silica (heavy line) and the sample after myoglobin adsorption (fine line).

In FTIR spectra, the broad peak at around 3500 cm^−1^ was assigned to the stretch vibration of N–H (Figure 4). Three absorbance peaks in the range of 2800–3000 cm^−1^ were associated with stretching and bending vibration of C–H. Two negative peaks at 2200–2400 cm^−1^ were attributed to the contribution of CO_2_ in the environment. The absorption peak at 1630 cm^−1^ might be due to bending vibration of the adsorbed water molecules. The formation of polysiloxane was confirmed by the Si-O-Si band at 804 cm^−1^. The incorporation of the amino group was verified by symmetric –NH_2_ bending vibration at 1515 cm^−1^ [17].

### 2.2. Assignment of UV/VIS and FTIR Spectra of Amino-Functionalized Mesoporous Silica after Adsorption Myoglobin

The isoelectric point of myoglobin is 7.0 [20]. The isoelectric point of amino-functionalized mesoporous silica synthesized by the co-condensation synthesis increases in the range of 7.9~8.7 with the increasing molar composition of APTES in the range of 0~0.2 [23]. Because the maximum molar composition APTES (0.2) was used in the synthesis, the isoelectric point of amino-functionalized mesoporous silica was 8.7. The adsorption process was carried out at pH 7.5 in this experiment. At pH 7.5, myoglobin carried net negative charge while amino-functionalized mesoporous silica presented net positive charge. The electrostatic attraction between the amino-functionalized mesoporous silica and myoglobin could facilitate the adsorption of myoglobin.

After adsorption, three absorbance peaks in the range of 2800–3000 cm^−1^ presented band broadening, which was associated with the contribution of the methylene of myoglobin (Figure 4). Amide I at 1640 cm^−1^ and amide II at 1548 cm^−1^ band were assigned to α-helix-rich protein, which verified the presence of myoglobin in amino-functionalized mesoporous silica.

**Figure 5:**
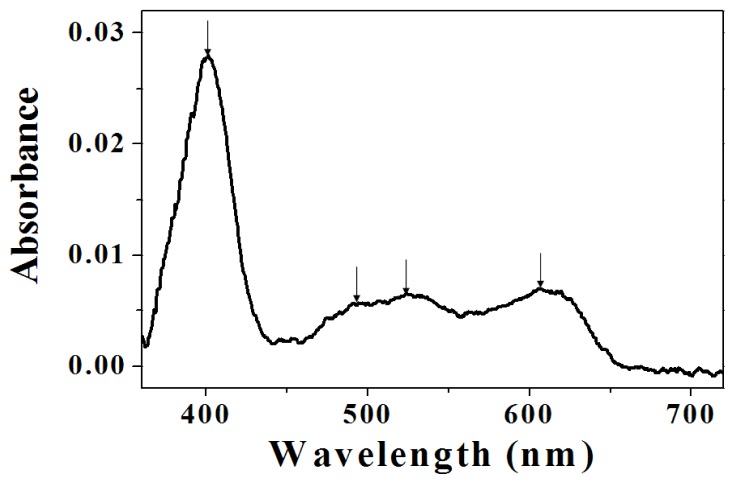
UV/VIS (Ultraviolet–visible) spectroscopy of myoglobin adsorbed on aminofunctionalized mesoporous silica.

In UV/VIS spectroscopy, the UV absorbance of amino-functionalized mesoporous silica acted as the baseline (Figure 5). The intensive peak at around 400 nm was assigned to the soret-band of porphyrin [24]. The two weak peaks at around 500 nm and one weak peak at 600 nm were assigned to the Q-band of porphyrin, respectively [25]. These peaks exhibited characteristic absorption of heme of myoglobin. The UV/VIS spectroscopy verified that myoglobin was successfully adsorbed into amino-functionalized mesoporous silica.

### 2.3. Evaluation of Peroxidative Activity of Myoglobin

**Figure 6:**
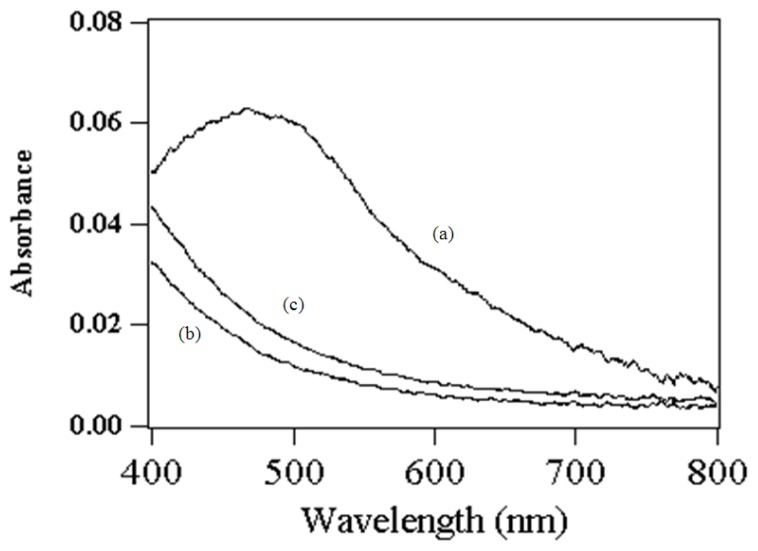
UV/VIS spectra of product produced by adsorbed myoglobin (**a**); amino-functionalized mesoporous silica (**b**) and amino-functionalized mesoporous silica linked by lysozyme without myoglobin (**c**).

After adsorption, lysozyme was covalently tethered to the surface of glutaraldehyde activated amino-functionalized mesoporous silica. The aldehyde group of the glutaraldehyde molecule connected with the amino group of amino-functionalized mesoporous silica and lysozyme through Schiff’s base reaction [26]. As can be seen from Figure 6, the absorbance peak of the produced bisazobiphenyl appeared at 472 nm, which was in accordance with the result of Claiborne and coworkers [27], suggesting that the adsorbed myoglobin still exhibited peroxidative activity after covalent attachment of lysozyme.

### 2.4. Antibacterial Property of Lysozyme

**Figure 7:**
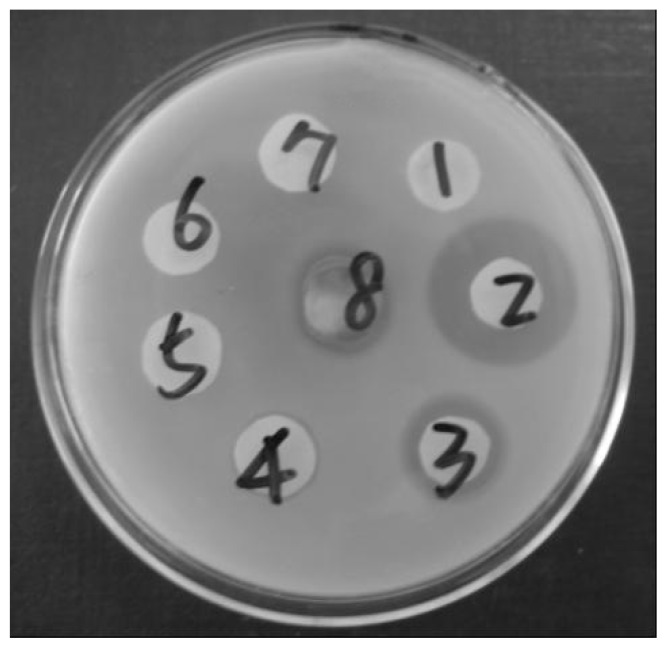
Antibacterial assay of sample: (**1**) amino-functionalized mesoporous silica adsorbed myoglobin; (**2**–**7**) the supernatant obtained from washing the final immobilized enzyme over four times; (**8**) the final immobilized enzyme.

The antibacterial activity of immobilized lysozyme was determined. As shown in Figure 7, there was no antibacterial circle for sample No. 1, suggesting that amino-functionalized mesoporous silica adsorbed myoglobin had no antibacterial activity. After the lysozyme was covalently linked to the amino-functionalized mesoporous silica, the immobilized enzyme was washed with buffer and the antibacterial activity of the washing supernatant was detected. No activity of lysozyme could be found in the supernatant after washing over four times, suggesting that the free or adsorbed lysozyme was totally removed. The bacteriostatic circle of the final immobilized enzyme (sample No. 8) clearly verified that covalently tethered lysozyme still possessed antibacterial activity (Figure 7).

The final immobilized enzyme was suspended in the buffer (pH 9.0 Gly-NaOH) for two hours and centrifuged. No protein could be detected in the supernatant, which demonstrated that the leaching of myoglobin did not occur. The final immobilized enzyme exhibited the activity of both myoglobin and lysozyme (Table 1) and the myoglobin/lysozyme ratio in the final immobilized enzyme was 0.624. According to the Betancor’s report, the monomer form of glutaraldehyde was grafted on the surface of amino-functionalized mesoporous silica when the concentration of glutaraldehyde applied was 5% (*v*/*v*) [28]. In this experiment, the concentration of glutaraldehyde used was 2.5% (*v*/*v*) so that the smaller spacer arm (monomer) was presented on the surface of amino-functionalized mesoporous silica. The shorter spacer arm (monomer) should offer a higher rigidity which was propitious to avoiding the leaching of the adsorbed myoglobin.

## 3. Experimental Section

### 3.1. Materials

All commercially available reagents were as follows: Pluronic P123 (EO_20_PO_70_EO_20_, *M*_av_ = 5800) (Aldrich); Tetraethoxysilane (TEOS) (Aldrich); 3-Aminopropyltriethoxysilane (APTES) (Fluka); Myoglobin (95%–100%, Sigma); Lysozyme (Amresco). All chemicals were used as received.

### 3.2. Experimental Procedures

#### 3.2.1. Synthesis of Aminopropyl-Functionalized SBA-15 Mesoporous Silica

The preparation of aminopropyl-functionalized mesoporous silica followed Soofin Cheng’s work [17]. SBA-15 was prepared according to the classical synthesis route [12].

#### 3.2.2. Characterization of the Amino-Functionalized Mesoporous Silica Materials

N_2_ adsorption-desorption isotherms was measured using Autosorb-1C (Quantachrome) at 77 K. The measurement process followed Soofin Cheng’s report [17]. Fourier transform infrared (FTIR) was carried out with a Thermo Nicolet 5700 spectrometer with a resolution of 4 cm^−1^ through the KBr method. Thermogravimetric (TG) analysis was carried out on a Q500 thermogravimetric analyzer (TA) with a heating speed of 10 °C/min under balance air at a flow of 40 mL/min and carrier air at a flow of 60 mL/min.

#### 3.2.3. Adsorption of Myoglobin

Amino-functionalized mesoporous silica (80 mg) was added to 12 mL of myoglobin solution (1 mg/mL, prepared in pH 7.5 PBS buffer) at 4 °C with slow stirring for one hour. Unless otherwise indicated, the buffer mentioned in this manuscript is pH 7.5 PBS buffer. After centrifuging, the resulting sample was washed with several times by the buffer. Until the value of the absorbance of the supernatant at 280 nm was less than 0.02, the operation of the washing was stopped. The sample was stored in the buffer.

#### 3.2.4. Characterization of Amino-Functionalized Mesoporous Silica Materials after Adsorption

After centrifuging, the resulting sample was dried at room temperature to gain dry power. Dry sample was detected using Shimadzu UV-3600 spectrometer and measured using Nicolet 5700 FTIR spectrometer with a resolution of 4 cm^−1^ through the KBr method.

#### 3.2.5. Measurement of Peroxidase Activity after Adsorption

The assay of peroxidase activity was perofrmed by modification of the method of Sil [29].The reaction system containing 600 μL of suspension (<0.05 g/mL), 80 μL of H_2_O_2_ (30%) and 3.6 mL of 0.002% o-dianisidine solution (prepared in buffer) was established, and then the reaction was conducted at 37 °C in 2 min. After centrifugation, the absorbance of the supernatant at 450 nm was recorded. For another parallel reaction system, the reaction mixture was dried at room temperature overnight. Dry sample was measured using the Shimadzu UV-3600 spectrometer. One unit of myoglobin activity was defined as the amount of enzyme to produce 1.0 μmol of oxidized product of o-dianisidine per min in defined condition.

#### 3.2.6. Glutaraldehyde Coupling Procedure

According to the report of Betancor *et al*., 350 mg of amino-functionalized mesoporous silica-myoglobin composition was added into 4 mL of glutaraldehyde (2.5%, *v*/*v* aqueous solution), and then the mixture was stirred at room temperature for two hours [26]. After centrifuging, the resulting sample was washed with several times by distilled water. After centrifuging, the precipitation was added into 1 mL of lysozyme solution. Then, this system was incubated in water bath at 25 °C for two hours. After centrifuging, the sample was washed with several times by buffer. The resulting sample was stored in the buffer.

#### 3.2.7. Enzymatic Activity Assay

After adsorption and covalent binding procedure, the peroxidase activity of myoglobin and the antibacterial activity of lysozyme were measured, respectively.

##### 3.2.7.1. Assay of Myoglobin

The experimental operation was the same as that of the procedure depicted in the Section of 3.2.5.

##### 3.2.7.2. Assay of Lysozyme

The resultant sample was repeatedly washed by a buffer. After centrifuging, the supernatant in every washing cycle was collected, respectively. After repeated washing, the precipitation was immersed in the buffer overnight. After centrifuging, the supernatant and the precipitation were collected on second day, respectively. Finally, all collections were used to infiltrate filter paper. Infiltrated filter paper was incubated in solid culture medium containing Micrococcus lysodeikticus at 37 °C for 18 h.

After washing, the immobilized lysozyme was suspended in buffer and allowed to strand for 10 min. The lysozyme activity was assayed by the reported procedure [30]. Micrococcus lysodeicticus was cultured on Difco nutrient agar for 48 h. The bacteria were washed by buffer to remove the agar. The bacteria were diluted by buffer to obtain an optical density of 1.3 at 450 nm. Immobilized lysozyme (1 mL) was added to 9 mL of diluted bacterial solution at 37 °C. The aliquot of the reaction mixture (1 mL) was taken at 30 s interval and then centrifuged at 1500*g* for 1 min. The UV absorbance of the supernatant at 450 nm was monitored by Shimadzu UV-2550 spectrometer. One unit of lysozyme activity was defined as the amount of lysozyme to cause a decrease in absorbance at 450 nm of 0.001 per min under these conditions.

The loading amount of immobilized enzyme (myoglobin or lysozyme) was quantified according to the difference between the total amount of enzyme added to the immobilization system and that recovered in the pooled supernatant and washing solutions. The protein content of the enzyme solutions was determined using Bradford method [31]. Bovine serum albumin was used as standard.

## 4. Conclusions

By using aminopropyl-functionalized SBA-15 mesoporous silica as support, a novel bifunctional bioreactor was obtained. The bifunctional bioreactor not only avoided the leaching of myoglobin in amino-functionalized mesoporous silica but also exhibited the enzymatic activity of myoglobin and lysozyme.

## Figures and Tables

**Table 1:** The protein loading and the activity of the final immobilized enzyme.

	Myoglobin	Lysozyme
Protein loading (mg/g)	88.5 mg/g	122.4 mg/g
Enzyme activity (U/g)	1398 U/g	1.32 × 10^6^ U/g
